# The Memoirs of Dr. Marshall Hall

**Published:** 1861-11

**Authors:** 


### Selections.

### THE MEMOIRS OF DR. MARSHALL HALL.

*Reprinted from the Lancet.*

So long ago as 1841, whilst the illustrious English physiologist, Marshall Hall, was battling with the Royal Society at home, for so much of recognition of his labors as may be implied by publication in the "Philosophical Transactions," the writer of this article heard Flourens, in a public discourse at the Jardin des Plantes, rank him with Harvey and Bell as one of a constellation of discoverers, which did not admit a fourth. It has been well said, that foreigners are, in some sense, a contemporaneous posterity. They survey the achievements of living genius uninfluenced by the disturbing passions that local proximity and personal competition engender. The judgment of Flourens is certainly, in this case, but an anticipation of that of after ages. Although those who best knew Marshall Hall could not help feeling the liveliest sympathy for the mental sufferings which he endured during the promulgation and establishment of his discoveries, and however bitter may be the self-accusing reproaches of those who combatted his doctrines, denying their truth with invective that savored more of personal envy than of philosophical caution, the world at large will rejoice that in this instance, as in many others, opposition served to rouse to greater exertion and to the production of more various and more logical proofs. In listening to the accusations of infirmity of temper, of over-sensitiveness, so often and so pertinaciously urged by his opponents against Marshall Hall, one would think that these qualities barred all right of fame ; that a discoverer has no business to have human resentments ; that a sense of wrong endured deprived him of all claims to gratitude for benefits which he may truly be said to have showered upon mankind. Have these opponents ever reflected that, in dwelling upon the sensitiveness of Marshall Hall under unworthy treatment, they are uttering their own condemnation? It is a confession of personal motives. The infirmity of Hall—if infirmity it were—was that of a noble mind ; it was, like the keen sagacity and absorbing love of truth that led to his discoveries, an attribute of the same intellectual and moral character. He who has made, and who knows he has made, a discovery fraught with benefit to mankind, cannot be indifferent to the reception of his gift—that is, if he be an honest man, not only great, but good. And this, beyond all cavil, Marshall Hall pre-eminently was. The greatness of the man, as a physiologist and physician, will not now be denied. No biography could prove this so effectually as his own writings. The value of his contributions to science we all acknowledge. But all cannot know what amiable, what pure and lofty sentiment adorned his private character. To show how well, deep and unaffected piety and domestic virtues can consort with the most exalted intellect and the most persevering pursuit of scientific truth, we take to be the chief and cherished object of the memoirs of Dr.

Marshall Hall, edited by his widow. To exhibit so admirable an illustration of this union of moral worth and intellectual grandeur is to confer an obligation upon society, hardly second to that conferred by the promulgation of the most useful discoveries. This task could by no one be so fitly or so completely executed as by Mrs. Marshall Hall. Not only because the opportunities of the wife must necessarily be more extensive than could fall to the lot of any other person, but also because in this instance the wife possesses the rarer qualifications of remarkable intelligence and superior literary capacity. We cannot avoid heartily congratulating the public upon the course of events which resulted in the undertaking of this biography by Mrs. M. Hall, instead of by "an able physiologist." We entirely approve the judgment of the friend who, being consulted upon the question of passing the book through editorial hands, replied that "it would injure the proposed biography to place it in the hands of any professional author or writer, with a view to having it dressed up. It would thus lose more in freshness and interest than it would gain in the way of polish." Those who read the work —will not be of opinion that the author needed much literary assistance. The narrative flows freely and perspicuously, lighted indeed by the warmth of affection, but not the less chastened by never-failing good sense.

Years ago there was published in this journal a short biography of Marshall Hall. It is needless to recapitulate in chronological order the story of his life. It is one, indeed, full of interest and of instruction, and will long be referred to as an example of the triumph of industry,—which some say is the essential quality of genius,—prompted by an ardent passion for the investigation of truth. Hall was a self-taught man. His early education was very imperfect. He applied himself with rigorous perseverance to the task of achieving a mastery over the Latin language, and succeeded so well, although the time devoted to it was subtracted from medical study, that in passing his examination at the College of Physicians he was specially complimented by Sir Henry Halford upon the elegance of his Latin. This example may serve to encourage many a student, fretting under a sense of deficiency in this respect, and perhaps regarding the new educational curriculum of the Medical Council as a serious bar to his entry into the profession, to rely upon himself, and to overcome by his own will that difficulty which early neglect had thrown in his way. He may be assured that when he has thus acquired the language he will find his faculties better trained for the career of observation and reflection before him than if the Accidence had been drummed into him by the ordinary method.

Marshall Hall was one of the few men who, having commenced their professional career in the provinces, have made a successful migration to the metropolis; and still more remarkable will this success appear when it is remembered that he at no time held the office of physician to a London hospital. His brilliant scientific and successful professional labors were all due to his own energies and sterling character; he owed nothing to the adventitious circumstances which are commonly and truly held to be essential to success. Yet no man was better fitted to shine as a clinical teacher; his faculty in diagnosis had been early and continuously cultivated; his power of demonstration to others was lucid and impressive in the extreme; as a lecturer few men have ever surpassed him in the accuracy of the knowledge he imparted, perhaps none ever equalled him in the rich suggestion of fertile thought. The student who had listened to one of his discourses went away glowing with the enthusiasm of a newly-kindled and new-seeing love of his profession; he felt that he had profited far beyond the acquisition of the particular facts of doctrines imparted to him—he felt new powers within himself, and saw before him fields for the exercise of his faculties hitherto undreamed of. Yet this man, before whom all the teachers of his day must have paled, was an unsuccessful candidate for the chair of Pathology at University College. This is not indeed the only instance of a man being rejected because he was too good; but it is not the less a matter of regret that the institution which had been honored by the teaching of Charles Bell, should have wilfully lost the irretrievable distinction of having numbered amongst its professors two out of the three great historical names in Pathology. Marshall Hall had an intense love of teaching: to the loss of personal comfort, to the injury of his professional income, and ultimately to the sacrifice of his health, he pursued this vocation. For some considerable time he lectured twice in the day at two schools situated at two extremes of the town. The result of this strain upon his physical powers was to lay the foundation of that disease in his throat which carried him to his grave. A routine reader of dry excerpts might not feel the exertion; but a teacher like Hall, giving earnest utterance to exuberant thoughts, seeking to animate his hearers with that love of research which burned within himself, could not pursue this course with impunity. The flame consumed the lamp.

But we are indulging too far in our own reflections, and are neglecting the "Memoirs." The narrative is full of points of attraction. The picture it reveals of domestic affection and happiness, of the tender husband and of the watchful father, is one of touching beauty. Passages of travel and description abound, for Hall was an ardent admirer and keen observer of Nature in all her works.

Here is a good story well told:

"Dr. Hall had for a friend and near neighbor, at Nottingham, Archdeacon Wilkins. This gentleman was engaged in the authorship of a well-known work called 'Body and Soul,' and had sent to Dr. Hall some of the proof-sheets for perusal. Dr. M. Hall having retained these longer than was convenient, Dr. Wilkins facetiously wrote a note to the following effect: 'Dear Dr. Hall,—Do send me back my body and soul; I cannot exist any longer without them.' The note was given to Dr. Hall's man-servant, whose curiosity led him to press its sides so as to be able to read the contents; for it was long before the modern fashion of envelopes. He rushed, aghast, into the kitchen, exclaiming, 'Cook, I cannot live any longer with the Doctor!' 'Why, what's the matter?' 'Matter enough,' replied the man; 'our master has got Dr. Wilkins' *body and soul*, and I have too much regard for my character to stay where there are such goings on!'"

Dr. Hall's first work was on Diagnosis, but his reputation as a practical physician was first made by his work on the Diseases of Women, and in the early part of his career his practice lay chiefly in this department; but "when," he says, "it became publicly known that my attention was directed to the nervous system, I gradually lost sight of the female disorders." His commentary upon Specialism is striking. "A physician in London," he remarks, "may have a reputation for knowing *one* thing well, *or* for knowing *all;* but he cannot have a reputation for knowing *two.* It is singular how steady my income was during this change of practice."

But other duties now compel us to break the chain that has long enthralled our attention to this book. We recommend it not alone to the medical practitioner and student, but to the general public. As a narrative, it is more interesting than a novel; as the memorial of a great English Worthy, it will be perused and preserved with pride; as an incentive to honorable toil and the practice of virtue, the story of the rise of Marshall Hall, deserves to take rank amongst the most favorite tales of the triumphs of genius.

All this is well told. Every page reflects honor upon the dead, and credit upon the author. Some critics may urge, that another might have produced a better biography of the physiologist than his wife. We doubt it. It may be true that we lose the record of many things which his wife could not know, or could not tell. But, on the other hand, we gain a richer and nearer insight into the man than any one else could possibly supply. The best testimony to the fitness of Mrs. Hall for the task lies, we conceive, in the very objection that is made to her. So well has she acquitted herself, that we close her book, not wearied of her subject, but stimulated by the desire for further details. So well has she sustained our interest in Marshall Hall, that we long to learn more of him. Some great men have fallen wonderfully in public esteem when their lives have been written; they have been crushed, as it were, under the dead weight of their biographies. Not so with Marshall Hall. Already enrolled by the acclamation of the scientific world, amongst the most illustrious of those who have benefitted mankind by their discoveries, his fame derives fresh glory from this simple story of his life, which reveals to us, a man of unostentatious piety, of warm domestic affections, and of sterling moral worth.

